# Supplementary material for: Structures and function of a tailoring oxidase in complex with a nonribosomal peptide synthetase module
Source: Nat Commun. 2022 Jan 27;13:548. doi: 10.1038/s41467-022-28221-y (PMC8795117; doi:10.1038/s41467-022-28221-y)
Supplement: Supplementary file 1 — Supplementary Information [file 41467_2022_28221_MOESM1_ESM.pdf]

Supplementary Information for:

Structures and function of a tailoring oxidase in complex with a nonribosomal peptide synthetase module

Camille Marie Fortinez[1,2], Kristjan Bloudoff[1,2], Connor Harrigan[1,2], Itai Sharon[1,2], Michael Strauss[2,3] & T. Martin Schmeing[1,2]

	**BmdC** **(PDB: 7LY6)** Wavelength = 0.979 Å	**Proteolyzed structure BmdB:BmdC** **(PDB: 7LY5)** Wavelength = 0.979 Å	**BmdBM$_2$:BmdC** **(PDB: 7LY7)** Wavelength = 0.979 Å
**Data collection**			
Space group	I2$_1$3	R32	P6$_5$22
Cell dimensions<br>a, b, c (Å)<br>α, β, γ (°)	 188.15, 188.15, 188.15<br>90, 90, 90	 85.524, 85.524, 415.19<br>90, 90, 120	 149.96, 149.96, 319.55<br>90, 90, 120
Resolution (Å)	94.08-2.73 (2.86-2.73)	46.3-2.5 (2.54-2.5)	82.36 – 3.8 (4.1-3.8)
Rpim	0.0.088 (0.898)	0.033 (0.261)	0.150 (0.678)
I/σI	23.5 (1.6)	29.5 (2.6)	4.5 (1.5)
CC$_{1/2}$	99.9 (35.4)	99.9 (93.3)	99.6 (47.3)
Completeness (%)	99.9 (100)	99.7 (99.9)	100 (100)
Redundancy	9.2 (8.7)	11.7 (10.4)	23.3 (21.5)
**Refinement**			
Resolution (Å)	94.07-2.73 (2.82-2.73)	46.31-2.50 (2.56-2.50)	49.28-3.80 (3.88-3.80)
No. reflections	29493	20713	21691
Rwork/Rfree	0.1719/0.1944	0.2286/0.2551	0.2979/0.3259
No. atoms (non-hydrogen)			
Protein	2599 atoms	BmdC (1410 atoms)<br>BmdB (2138 atoms)	BmdC (2664 atoms)<br>BmdB (7338 atoms)
Ligand/ion	FMN (31 atoms)<br>Glycine (5 atoms)<br>Phosphate (5 atoms)	FMN (31 atoms)	FMN (31 atoms)<br>YOA (49 atoms)
Water	101	119	0
B-factors			
Protein (mean)	70.33	65.77	183.05
Ligand/ion	72.57	76.25	158.54
R.m.s deviations			
Bond length(Å)	0.0122	0.007	0.003
Bond angles (°)	1.247	0.888	0.740
Clashscore	1.75	3.69	9.51
Ramachandran favored	96.57%	95.32%	93.00%
Ramachandran outliers	0%	0%	0.90%
Side chain rotamer outliers	0.00%	2.41%	0.65%

Supplementary Table 1: **Data collection and refinement statistics for X-ray crystallography structures**. The structures in this study were determined with data collected from a single crystal. The values in parentheses are for the highest-resolution shell.

	Full complex	Local refinement
EMDB accession #	23588	23587
Magnification	105,000x	105,000x
Voltage (kV)	300	300
Total micrographs	8,284	8,284
Total electron exposure ($e^- / Å^2$)	109	109
Frames	40	40
Exposure time (s)	4	4
Defocus range (μm)	-1 to -3	-1 to -3
Pixel size (Å)	0.855	0.855
Symmetry imposed	C1	C1
Final particles	122,038	117,491
Resolution (masked FSC = 0.143, Å)	4.2	3.8
Resolution range (Å)	3.6 to 10	2.4 to 10

Supplementary Table 2. Statistics for Cryo-EM data collection

	**$BmdBM_2$:BmdC (PDB: 7LY4)**
Model refinement resolution (Å)	3.8
RMS deviations	
Bond length (Å)	0.019
Bond angle (°)	1.747
Ramachandran plot	
Favoured (%)	97.68
Allowed (%)	2.11
Outliers (%)	0.20
Rotamer outliers (%)	0.23
Validation	
MolProbity score	1.13
Clashscore	2.76
C-beta outliers	0.14

Supplementary Table 3. Model refinement statistics of model from electron microscopy reconstruction. This was locally refined into the cryo-EM map found in the EMDB with accession code 23587.

Chemical Reagent	Commercial supplier
4- (2-hydroxyethyl)-1-piperazineethanesulfonic acid (HEPES)	Thermo Fisher Scientific (BPS310-100)
Adenosine triphosphate (ATP)	BioShop (ATP008)
Ammonium acetate	BioShop (ACE601)
Bacteriological tryptone	Thermo Fisher Scientific (211699)
Bis-Tris propane	BioShop (BTP501)
β-mercaptoethanol (β-ME)	BioShop (MER002)
Butanol (1-butanol)	BioShop (BTU001)
Calcium chloride dihydrate	BioShop (CCL302)
Chloroform	BioShop (CCL402)
Coenzyme A (CoA), trilithium dihydrate	BioShop (COA111)
Dithiothreitol (DTT)	BioShop (DTT001)
Ethylene Glycol	Sigma Aldrich (324558)
Ethylene glycol tetraacetic acid (EGTA)	Bio Basic (ED0077)
Flavin adenine dinucleotide (FAD), disodium salt hydrate	Sigma Aldrich (F6625)
Flavin mononucleotide (FMN), sodium salt hydrate	Sigma Aldrich (F2253)
Formic acid	Thermo Fisher Scientific (85178)
Glycine	BioShop (GLN001)
Guanidine hydrochloride	BioShop (GUA999)
Hexylene glycol (MPD)	Sigma Aldrich (112100)
HPLC water	Thermo Fisher Scientific (W5-4)
HPLC acetonitrile	Thermo Fisher Scientific (A998-4)
Imidazole	BioShop (IMD508)
Isopropylthio-d-galactoside (IPTG)	Thermo Fisher scientific (BP1755)
Kanamycin sulfate	Bio Basic (KB0286)
L-alanine	BioShop (ALA001)
L-cysteine	BioShop (CYS555)
L-tryptophan	BioShop (TRP100)

Magnesium chloride hexahydrate	BioShop (MAG510)
Methanol	Thermo Fisher scientific (A452)
Polyethylene glycol (PEG) 1500	Sigma Aldrich (86101)
Polyethylene glycol (PEG) 3350	Sigma Aldrich (202444)
Potassium chloride	BioShop (POC308)
Pyridoxal 5'-phosphate (PLP)	Sigma Aldrich (P9255)
Sodium chloride	Thermo Fisher scientific (S271-1)
Sodium citrate tribasic dihydrate	Sigma Aldrich (S4641)
Sodium dihydrogen phosphate monohydrate	BioShop (SPM306)
Succinic acid	Sigma Aldrich (398055)
Tris(2-carboxyethyl)phosphine (TCEP)	BioShop (TCE101)
Tris(hydroxymethyl)aminomethane (TRIS)	BioShop (TRS001)
Tryptamine	Sigma Aldrich (193747)
Yeast extract	BioShop (YEX401)

Supplementary Table 4. Complete list of commercial reagents used in this study in alphabetical order. The catalogue number of the reagents are indicated in parentheses.

Protein construct name	Primer name	Sequence (5’ -> 3’)	Source of amplified DNA	Parental plasmid	Rest-riction site	Resulting plasmid	Affinity tags
BmdC	bmdC-F	actgtgccatgggcgagcaaatcttt	*T. vulgaris* genome	pBacT	Nco1	pBacT_BmdC	N: His8
	bmdC-R	actgtggcggccgccccatcaatcttcaaac			Not1		C: CBP
BmdA	bmdA-F	actgtggaattcatacaaaaatttgcagttgtc	*T. vulgaris* genome	pBacTRev	EcoR1	pBacTR_BmdA	N: CBP
	bmdA-R	actgtggcggccgcgatgggatgtttacatgaa			Not1		C: His8
BmdB_PCP$_2$	BT2-F	aaaaaagcttttacgttgcatgcggcat	pBacTRev_BmdB[1]	pBacT	Nco1	pBacT_BmdB_PCP$_2$	N: His8
	BT2-R	aaaaccatggaatttgtcgctccccaagc			Not1		C: none
BmdB_A$_2$	BA2-F	aaaccatggaatacaacgcaacggctga	pBacTRev_BmdB	pBacT	Nco1	pBacT_BmdB_A$_2$	N: His8
	BA2-R	aaaagcggccgcttagcgatccaatttcccatt			Not1		C: none
BmdB_C$_3$	BC3-F	aaaaccatggaaccgcatgcaacgatcga	pBacTRev_BmdB	pBacT	Nco1	pBacT_BmdB_A$_3$	N: His8
	BC3-R	tgtggaattcggtatctgaaaatcaccgatcgtttgc			EcoR1		C: CBP
BmdB_A$_2$PCP$_2$	BAT2-F	aaaaccatggaatacaacgcaacggctgaa	pBacTRev_BmdB	pBacT	Nco1	pBacT_BmdB_A$_2$PCP$_2$	N: His8
	BAT2-R	aaaagcggccgcttacaacttcttaatcgtccgcg			Not1		C: none
BmdB$_{M2}$	BM2-F	aaaaccatggaagcagatctttcggaccgt	pBacTRev_BmdB	pBacT	Nco1	pBacT_ BmdB_M2	N: His8
	BM2-R	aaaagcggccgcttacaacttcttaatcgtccgcg			Not1		C: none

Supplementary Table 5. PCR primers. PCR primer sequences, source of amplified DNA, parental plasmids, restriction sites used and resulting plasmid names for cloning in this study. All affinity tags are cleavable with TEV protease.

Protein construct name	Primer name	Sequence (5’ -> 3’)	Parental plasmid	Resulting plasmid	Affinity tags
BmdB_S792A	S792A-F	gctttgggtggagaa**gcg**attaaagccttgcagattat	pBacTRev_BmdB	pBacT_BmdB_S792A	N: CBP<br>C: His8
	S792A-R	ataatctgcaaggctttaat**cgc**ttctccacccaaagc			
BmdB_S1820A	S1820A-F	gctttgggtggagat**gcg**atcaaggcgattcagg	pBacTRev_BmdB	pBacT_BmdB_S1820A	N: CBP<br>C: His8
	S1820A-R	cctgaatcgccttgat**cgc**atctccacccaaagc			
BmdC_R146E	R146E-F	cggacattattcaaaaacgc**ga**atcttgccgccggtt	pBacT_BmdC	pBacT_BmdC_R146E	N: His8<br>C: CBP
	R146E-R	aaccggcggcaagat**tc**gcgtttttgaataatgtccg			
BmdC_R146E_S292F	S292F-F	ttgaatgtggggttgtgtt**tt**attgggcacatgaatttc	pBacT_BmdC_R146E	pBacT_BmdC_R146E_S292F	N: His8<br>C: CBP
	S292F-R	gaaattcatgtgcccaat**aa**aacacaaccccacattcaa			
BmdB$_{M2_struct}$	M2St-F	cgcggacgattaagaagttggcggccgcagaga	pBacT_BmdB_M2	pBacT_BmdB_M2_struct	N: His8<br>C: CBP
	M2St-R	tctctgcggccgccaacttcttaatcgtccgcg			
BmdB_A$_2$-R1512D	R1512D-F	cgcaccgtcgag**ga**tcgggggatcacg	pBacT_BmdB_A$_2$	pBacT_BmdB_A$_2$-R1513D	N: His8<br>C: none
	R1512D-R	cgtgatcccccg**at**cctcgacggtgcg			
BmdB_A$_2$-R1513D	R1513D-F	ccgtcgagcgt**gat**gggatcacgatctg	pBacT_BmdB_A$_2$	pBacT_BmdB_A$_2$-R1513D	N: His8<br>C: none
	R1513D-R	cagatcgtgatccc**atc**acgctcgacgg			
BmdC_D225R	D225R-F	cttggtgttagtcaacaatatt**cg**tcaagtaatcaaggatgatcat	pBacT_BmdC	pBacT_BmdC_D225R	N: His8<br>C: CBP
	D225R-R	atgatcatccttgattacttga**cg**aatattgttgactaacaccaag			
BmdC_D231R	D231R-F	caatattgatcaagtaatcaaggacgtcatgagctgatcaatcaag	pBacT_BmdC	pBacT_BmdC_D2R	N: His8<br>C: CBP
	D231R-F	cttgattgatcagctcatgacgtccttgattacttgatcaatattg			
(BmdB_BamH1HindIII – cloning intermediate)	B-BamHind-F	cgctagatcatgtgggttct**ggatccgagagagaagctt**catttt gagaatatatcgttgaggttgg	pBacTRev_BmdB	pBacTRev_BmdB_BamH1HindIII	(N: CBP<br>C: His8)
	B-BamHind-R	ccaacctcaacgatatattctcaaaatg**aagcttctctctcggatc c**agaacccacatgatctagcg			
BmdB-BmdC(Ox)Link*Pt*	DelPtRRE-F	cattccaacaactcagcgt**atattgccgccaaggga**	pBacTRev_ BmdB-BmdCLink*Pt*	pBacTRev_ BmdB-BmdC(Ox)Link*Pt*	N: CBP<br>C: His8
	DelPtRRE-R	**tcccttggcggcaatat**acgctgagttgttggaatg			
BmdB-BmdC(Ox)Link*Bp*	DelBpRRE-F	aagcataaataagcaaagtaaaatagataag**atattgccgccaa ggga**	pBacTRev_ BmdB-BmdCLink*Bp*	pBacTRev_ BmdB-BmdC(Ox)Link*Bp*	N: CBP<br>C: His8
	DelBpRRE-R	**tcccttggcggcaatat**cttatctattttactttgcttatttatgctt			

Supplementary Table 6. Site-directed mutagenesis primers. Primer sequences, parental plasmids and resulting plasmid names for cloning in this study.

LC-method 1

Time (min)	Solvent A (%)<br>water + 0.1% formic acid	Solvent B (%)<br>acetonitrile + 0.1% formic acid	Flow rate (ml/min)
0.0	95	5	0.300
5.0	95	5	0.300
5.1	85	15	0.300
35.0	65	35	0.300
35.1	5	95	0.300
40.0	5	95	0.300
40.1	95	5	0.300
43.0	95	5	0.300

LC Method 2

Time (min)	Solvent A (%)<br>water	Solvent B (%)<br>10mM ammonium acetate, pH 6.6	Solvent C (%)<br>methanol	Flow rate (ml/min)
0.0	90	10	0	0.500
2.0	90	10	0	0.500
17.0	15	10	75	0.500
23.0	15	10	75	0.500
25.0	90	10	0	0.500
30.0	90	10	0	0.500

LC Method 3

Time (min)	Solvent A (%)<br>water + 0.1% formic acid	Solvent B (%)<br>acetonitrile + 0.1% formic acid	Flow rate (ml/min)
0.0	95	5	0.300
5.0	95	5	0.300
35.0	55	45	0.300
40.0	5	95	0.300
45.0	5	95	0.300
47.0	95	5	0.300
50.0	95	5	0.300

Supplementary Table 7. LC methods.

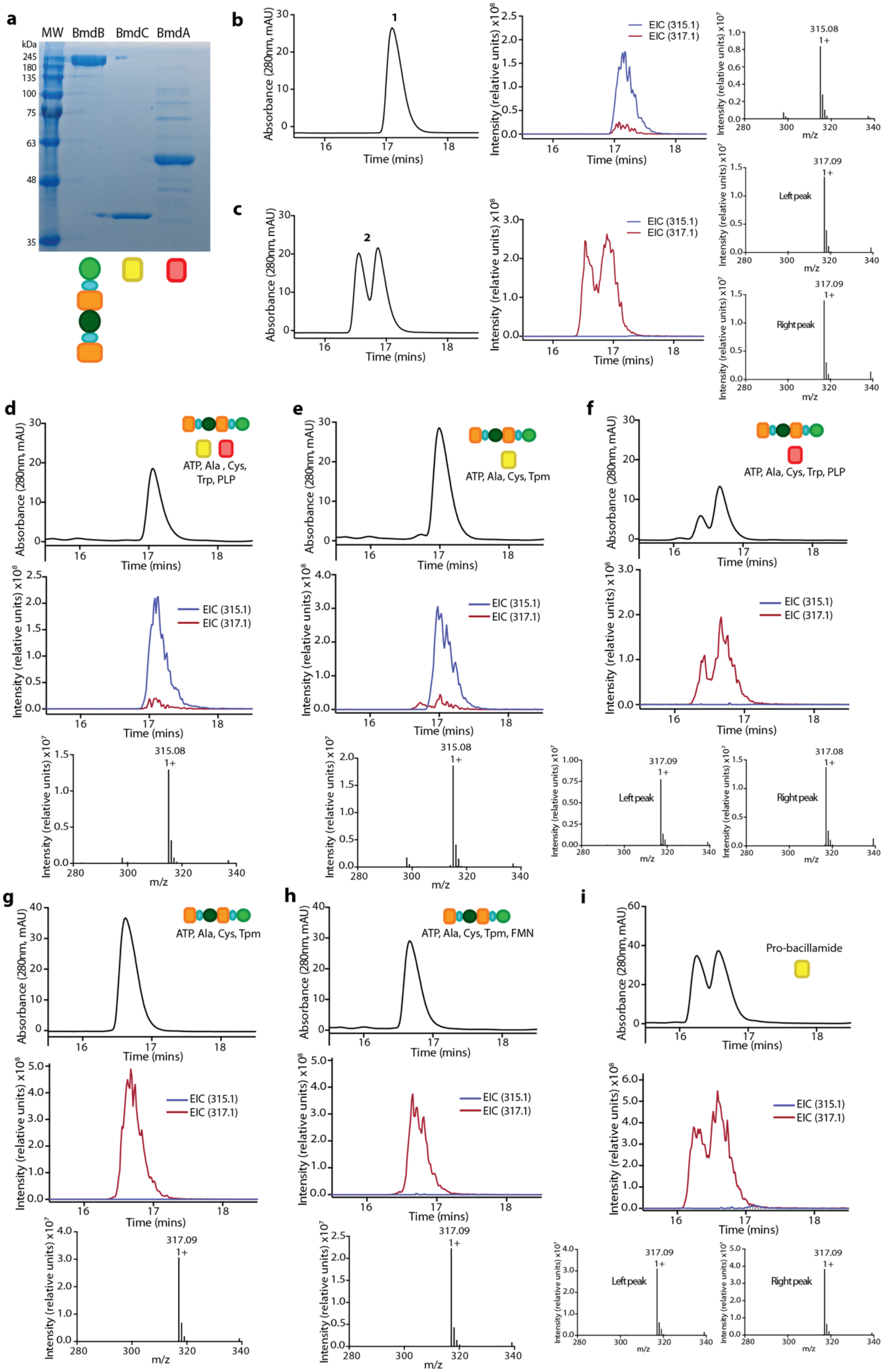

a
MW
BmdB
BmdC
BmdA
kDa
b
c
d
e
f
g
h
i
Absorbance (280nm, mAU)
Time (mins)
Intensity (relative units) x10⁸
EIC (315.1)
EIC (317.1)
Intensity (relative units) x10⁷
m/z
315.08
317.09
317.08
1+
Left peak
Right peak
ATP, Ala, Cys, Trp, PLP
ATP, Ala, Cys, Tpm
ATP, Ala, Cys, Tpm, FMN
Pro-bacillamide

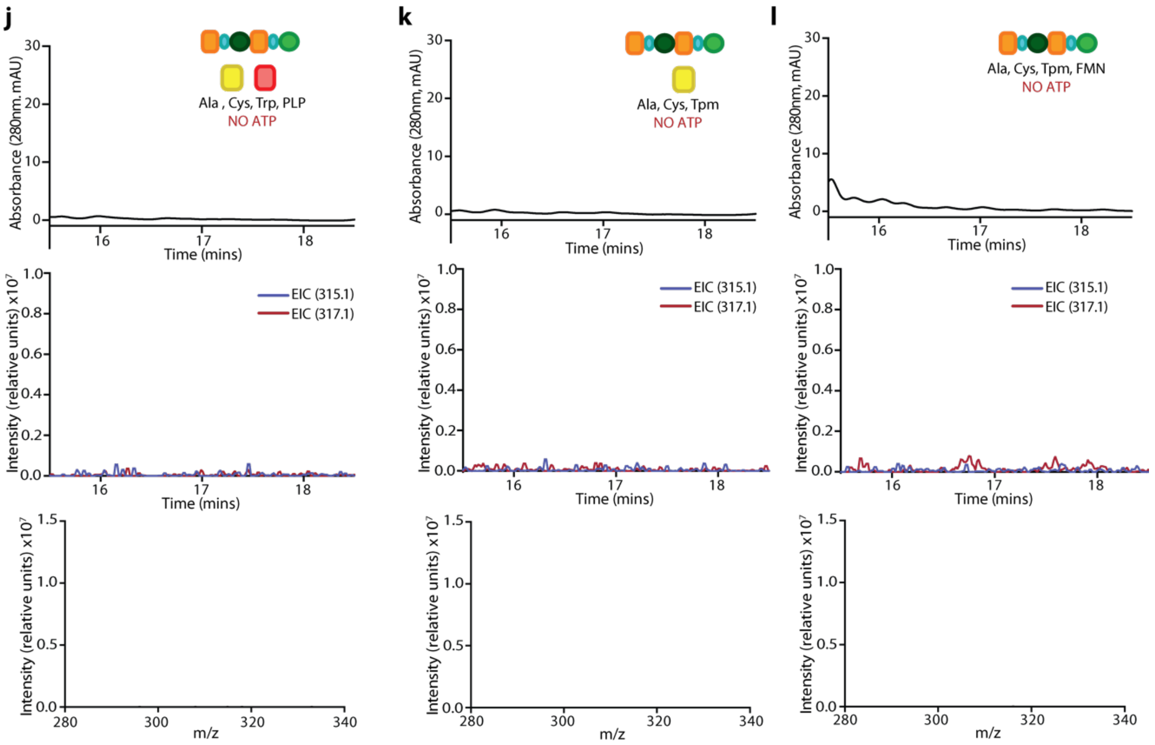

Supplementary Figure 1: **Bacillamide production by BmdA-C proteins *in vitro***.
a. An SDS gel shows purified BmdA, BmdB, and BmdC used. BmdA, BmdB, BmdC were purified at least three different times showing similar purity on an SDS gel. **b-c.** Mass spectrometry analysis of the bacillamide standards purchased from Zamboni Chemical Solutions. (b) Bacillamide D (**1**) and (c) pro-bacillamide (**2**). Throughout this figure, black curves represent absorbance at 280 nm wavelength, red curves represent extracted ion chromatographs of m/z = 317.1 and blue curves represent extracted ion chromatographs of m/z = 315.1. **d-i**. *In vitro* reaction combinations of BmdA, BmdB, BmdC and necessary substrates/cofactors demonstrating successful bacillamide production. (d). BmdA, BmdB, BmdC in the presence of necessary substrates and cofactors produces bacillamide D. (e) Replacing BmdA, PLP and Trp with tryptamine (Tpm) results in the production of bacillamide D. (f) BmdA and BmdB with the necessary substrates and cofactors results in pro-bacillamide. (g) Replacing BmdA, PLP and Trp with Tpm results in successful pro-bacillamide production. (h) Presence of exogenous FMN does not result in oxidized bacillamide D. (i) BmdC is unable to use pro-bacillamide as a substrate for oxidation. Reactions i-vi were each done in triplicate with similar results. **j-l**. Negative controls showing the lack of bacillamide production in the absence of ATP in reaction conditions involving (j) BmdA-C, (k) BmdB, BmdC, Tpm and (l) BmdB, Tpm with the necessary substrates and cofactors. Reactions j-l were each done in triplicate with similar results. Note that pro-bacillamide elutes as a double peak in the purchased standard (c, i) and in the BmdA-B reconstitution (f), likely because it exists as a pair of diasteromers with elution times of 16.3 and 16.7 min. Comparing the enzymatic synthesis of pro-bacillamide in the presence and absence of PLP and BmdA (f and g, respectively), suggests that the minor pro-bacillamide diastereomer (16.3 min) likely results from a racemization side reaction catalysed by the BmdA decarboxylase or free PLP coenzyme. Such promiscuity is common in PLP-dependent enzymes[2-6].

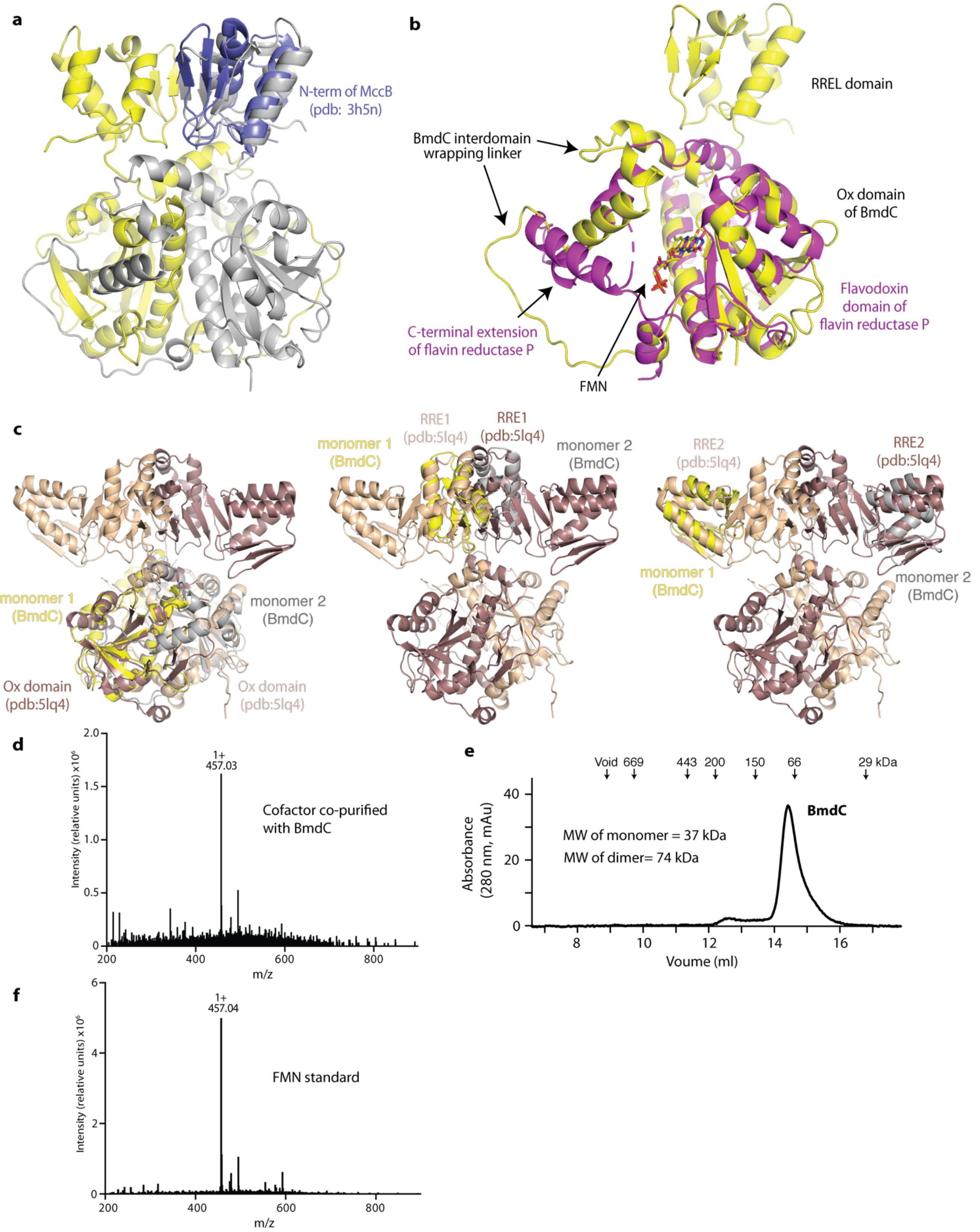

a
N-term of MccB
(pdb: 3h5n)
b
RREL domain
BmdC interdomain
wrapping linker
Ox domain
of BmdC
Flavodoxin
domain of
flavin reductase P
C-terminal extension
of flavin reductase P
FMN
c
monomer 1
(BmdC)
monomer 2
(BmdC)
Ox domain
(pdb:5lq4)
Ox domain
(pdb:5lq4)
RRE1
(pdb:5lq4)
RRE1
(pdb:5lq4)
monomer 1
(BmdC)
monomer 2
(BmdC)
RRE2
(pdb:5lq4)
RRE2
(pdb:5lq4)
monomer 1
(BmdC)
monomer 2
(BmdC)
d
Intensity (relative units) x10⁶
1+
457.03
Cofactor co-purified
with BmdC
m/z
e
Void 669 443 200 150 66 29 kDa
BmdC
MW of monomer = 37 kDa
MW of dimer= 74 kDa
Absorbance
(280 nm, mAu)
Voume (ml)
f
Intensity (relative units) x10⁶
1+
457.04
FMN standard
m/z

Supplementary Figure 2: Comparisons of the structure of BmdC with existing structures

a. Superimposition of the N-terminal portion of MccB[7] (light blue; pdb: 3h5n) and the RREL domain of BmdC shows both adopt a similar winged helix-turn-helix (wHTH) fold. **b**. Superimposition of the Ox domains of BmdC and reductase P shows contrasting topologies of wrapping linkers. In BmdC, the wrapping linker is N-terminal to that of the Ox domain, while for reductase P (magenta) [8], this extension is present at the C-terminus (pdb: 2bkj). **c**. Structural comparisons of BmdC and ThcOx[9] (pdb: 6grh). Left: Overlay of dimeric Ox domain of BmdC (yellow and gray) with the Ox domain of the dimeric ThcOx (beige and violet). Middle, right: RREL domains of BmdC overlaid on each of the two copies of RRE of ThcOx. **d**. Mass spectrometry of cofactor extracted from boiled BmdC **e**. Size exclusion experiments on wildtype BmdC shows that it is dimeric in solution. This was performed in triplicate with similar results. **f**. Mass spectrometry analysis of the FMN standard purchased from Sigma-Aldrich.

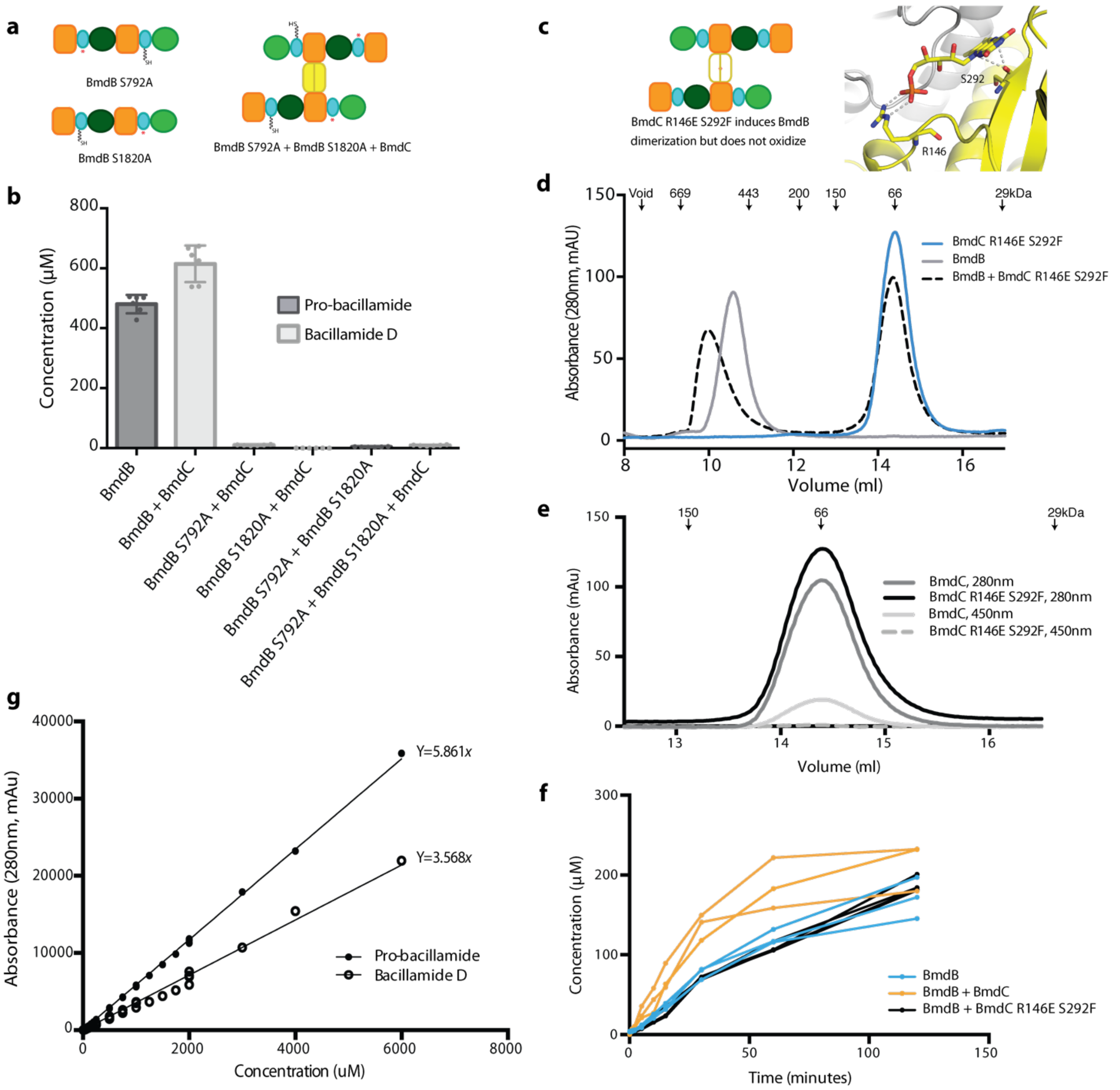

Supplementary Figure 3: **Dimerization of BmdB has no apparent catalytic advantage**.
a-b. Complementation experiments. (a) Schematic representation of the catalytically inactive mutants BmdB_S792A and BmdB_S1820A, which render PCP1 and PCP2, respectively, inactive by eliminating the pantetheine attachment point. (b) BmdB_S792A and BmdB_S1820A are unable to complement each other in the presence of wildtype BmdC *in vitro*. Each of the reactions was done in triplicate on two different days (n=6 independent reactions), and each replicate is shown. The central value represents the mean value, while the standard deviations to the mean are represented by the error bars. Concentrations of each respective bacillamide produced were normalized using the conversion described in panel (**g**). **c-f**. De-flavination experiments. (c) Schematic representation of the double mutant BmdC_R146E-S292F. (d) This double mutant can still dimerize BmdB successfully. (e) However, it is de-flavinated, as shown by the absence of signal at the 450nM wavelength measured during size exclusion chromatography. As a result, this BmdC mutant loses its function of oxidation. (f) Time course experiments reveal that pro-bacillamide production *in vitro* is no different from monomeric BmdB and dimeric BmdB induced by the BmdC_R146E-S292F mutant, and that the rate of bacillamide D production is faster than that of pro-bacillamide. This was done in triplicate (n=3 independent reactions) where each replicate is plotted. Concentrations of each bacillamide catalyzed were normalized using the conversion depicted in panel (**g**). **g**. Standard curve converting the pro-bacillamide and bacillamide D UV absorbance measurements (at the 280nM wavelength) to the respective concentrations. This standard graph was generated using standards purchased from Zamboni Chemical Solutions.

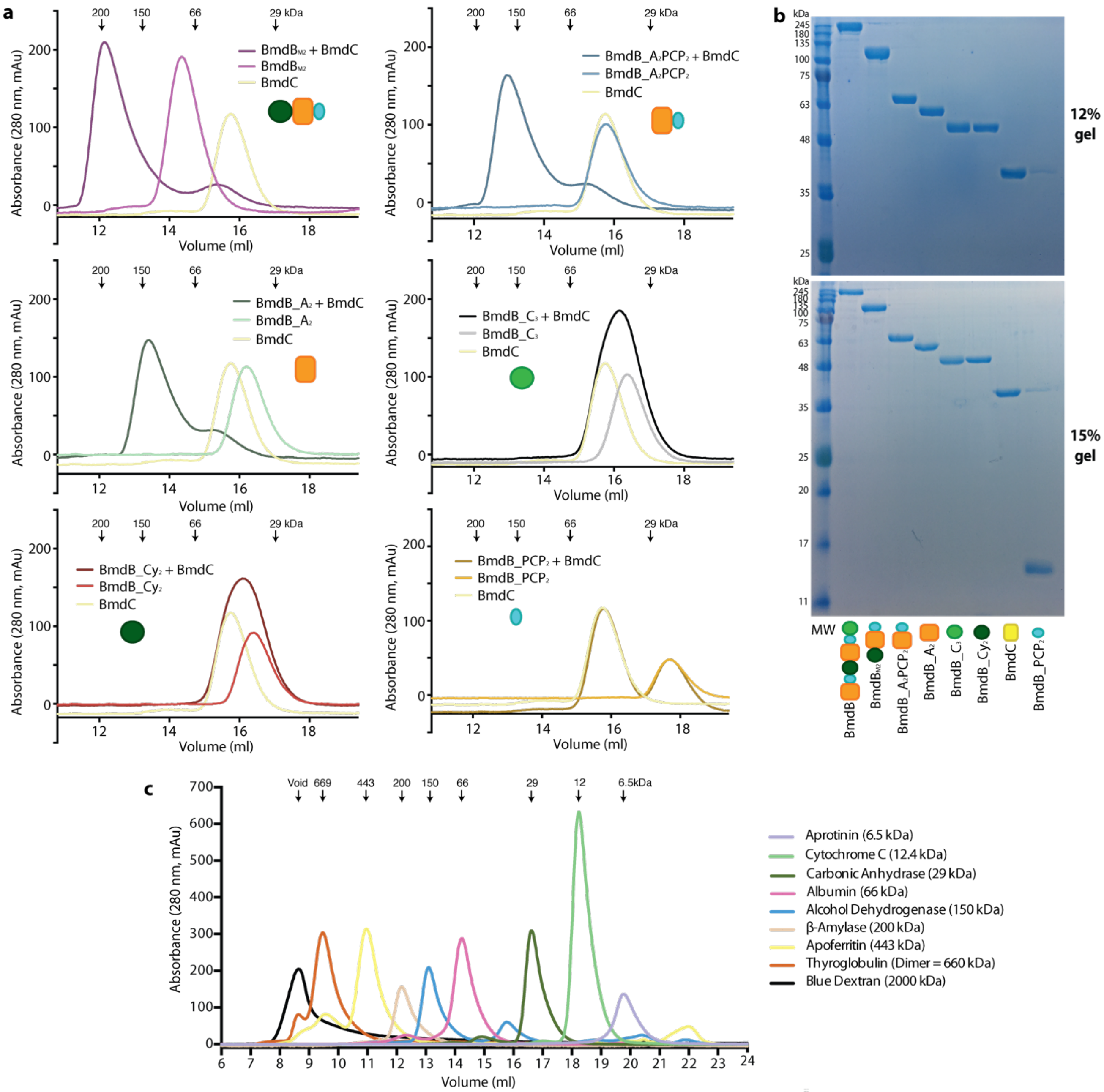

Supplementary Figure 4: **BmdC induces dimerization of BmdB through binding to the A2 domain**.

a. Size exclusion experiments of various constructs of BmdB in the presence and the absence of BmdC. Each protein (4μM) in a total volume of 500μL was used for these experiments. Dimerization induced by dimeric BmdC occurs only with BmdB constructs including the A_2 domain. BmdB_C_3, BmdB_Cy and BmdB_PCP_2 are unable to dimerize in the presence of BmdC at these given concentrations. **b**. SDS-PAGE gels of the constructs utilized for these gel filtration experiments showing the purity of the sample. All of the represented constructs have been purified at least three times showing similar purity on the SDS gel with the exception of BmdB_PCP_2 which has been purified once. **c**. Size exclusion experiments of protein standards applied on the Superdex 200 Increase 10/300 column.

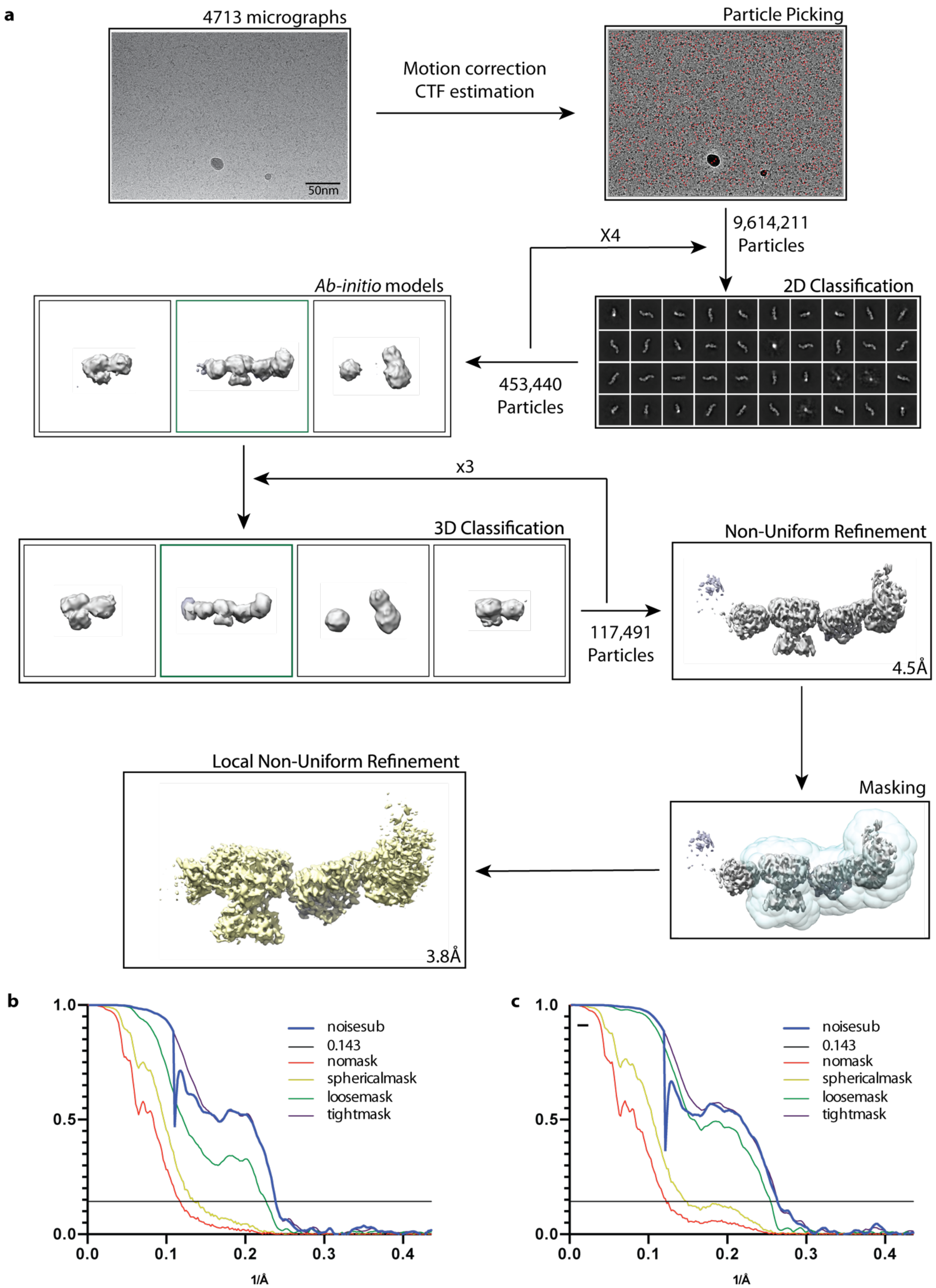

a
4713 micrographs
50nm
Motion correction
CTF estimation
Particle Picking
9,614,211
Particles
X4
2D Classification
Ab-initio models
453,440
Particles
x3
3D Classification
Non-Uniform Refinement
117,491
Particles
4.5Å
Masking
Local Non-Uniform Refinement
3.8Å
b
noisesub
0.143
nomask
sphericalmask
loosemask
tightmask
1/Å
c
noisesub
0.143
nomask
sphericalmask
loosemask
tightmask
1/Å

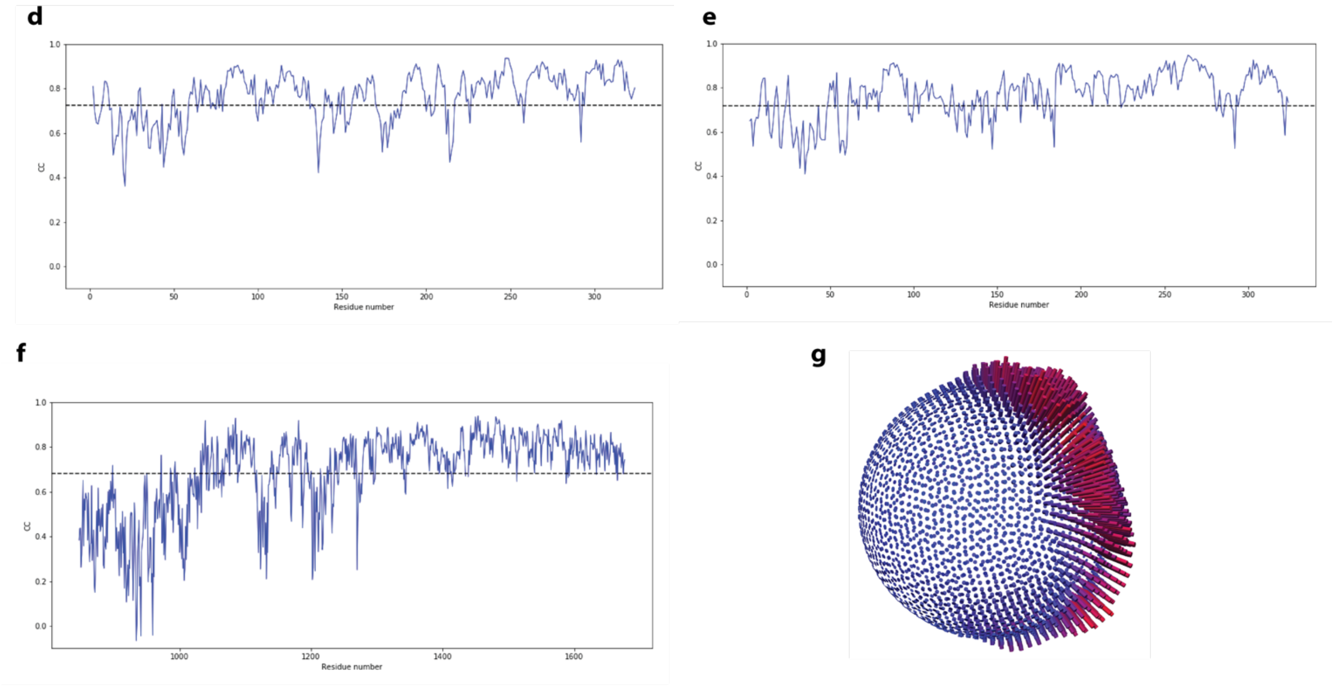

Supplementary Figure 5: Details of cryo-EM structure elucidation

a. Flow chart of the cryo-EM analysis and the structure elucidation of BmdB$_{M2}$-BmdC. **b-c**. FSC curves for both the full cryo-EM map of the BmdB$_{M2}$-BmdC dimeric complex (b) and the locally-refined map calculated using a mask around one copy of BmdB and one dimer of BmdC (c). The non-ideality of FSC curves are commonly observed in reconstructions which displays flexibility and preferred orientation. The dip at the 0.1 1/Å results from phase randomization. **d-e**. Per residue map-to-model CC plots of each BmdC monomer (**d-e**) and of the single BmdM2 monomer (**f**). **g**. Angular distribution plot of the BmdB$_{M2}$-BmdC dimeric complex. The relative number of particles is indicated by histogram height and color (blue – few particles; red – many particles).

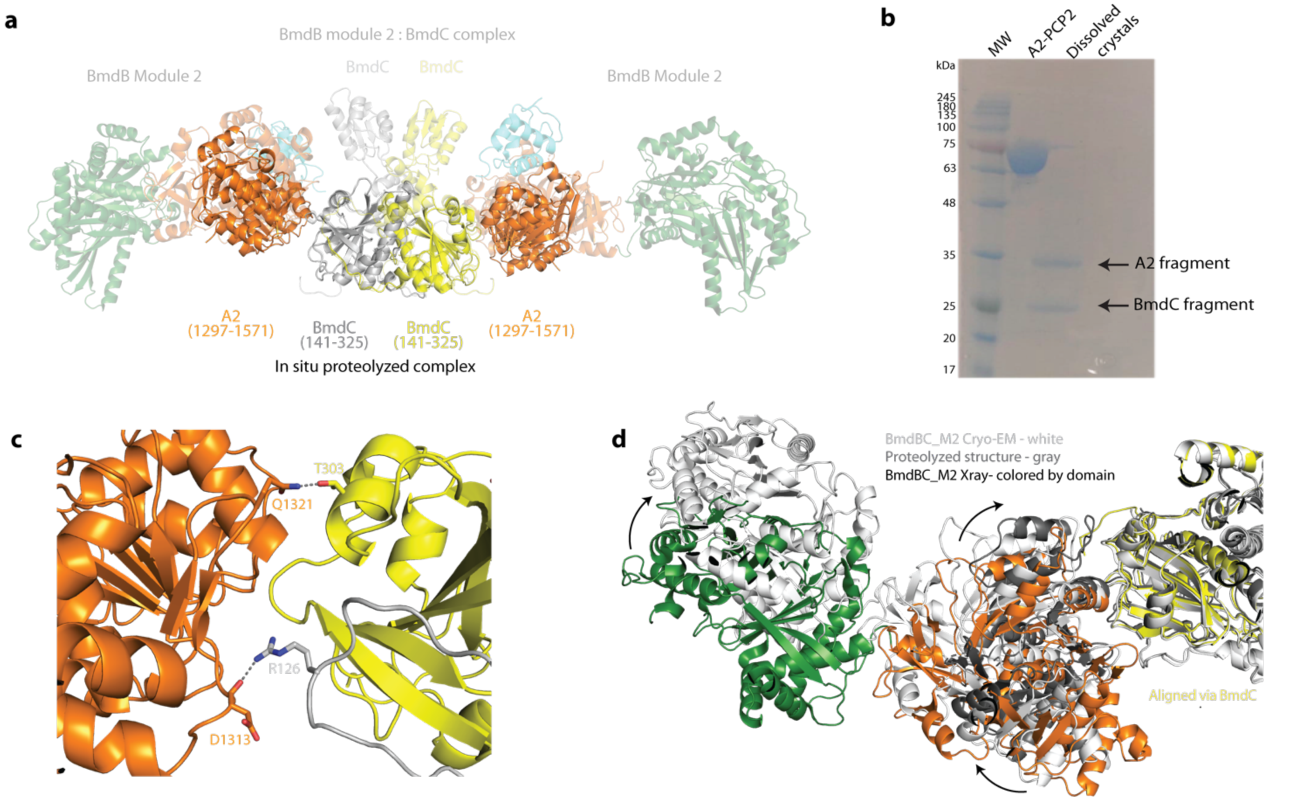

Supplemental Figure 6: Structural comparisons of all BmdB:BmdC complexes solved in this study.
a. Structural superimposition of the proteolyzed and the BmdB$_{M2}$-BmdC dimeric complexes solved by X ray crystallography to highlight the flexibility in the interface between the A$_2$ domain of BmdB and the Ox domain of BmdC. **b**. SDS-PAGE gel contrasting the original purified BmdB-A$_2$PCP$_2$ construct (~70 kDa) utilized for the crystallographic experiments with the buffer-dissolved crystals that lead to the crystallization and elucidation of the proteolyzed structure. The bands observed match in size with the remnants of BmdB_A$_2$ (~32 kDa) and the Ox domain of BmdC (~25 kDa) present in the solved proteolyzed structure. BmdB-A$_2$PCP$_2$ has been purified at least three times showing similar purity on the SDS gel. A total of three crystals were dissolved for the SDS sample. **c**. Additional hydrogen bond interactions present at the interface of the BmdB$_{M2}$-BmdC structure, not present in the proteolyzed structure of the complex. **d**. Overlay of all the structures solved of the BmdB:BmdC complex (either by cryo-EM or X-ray crystallography), to depict the shifts between the Cy$_2$:A$_2$ and the Ox:A$_2$ interfaces when aligned via BmdC.

a

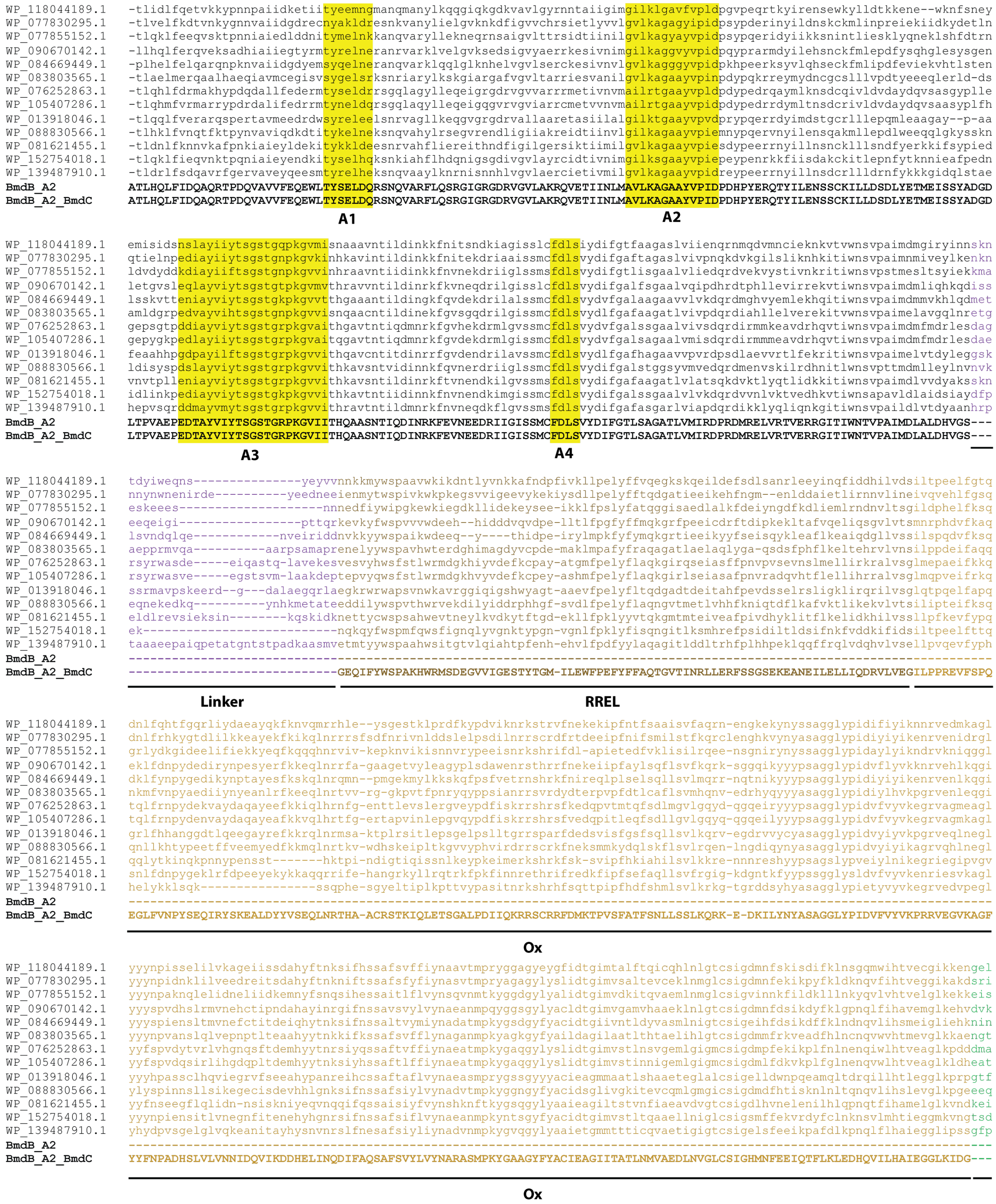
WP_118044189.1
WP_077830295.1
WP_077855152.1
WP_090670142.1
WP_084669449.1
WP_083803565.1
WP_076252863.1
WP_105407286.1
WP_013918046.1
WP_088830566.1
WP_081621455.1
WP_152754018.1
WP_139487910.1
BmdB_A2
BmdB_A2_BmdC
A1
A2
A3
A4
Linker
RREL
Ox

```
WP_118044189.1   tllpla------------------------------------------------------------kprkesqftntslrivmlsgdwipkelpakirsyapnakvvslggateasiwsi
WP_077830295.1   iipkesn------------rdiy-------------------------------------------geekfkledysslrlvllsgdwialdlprkikksfinsevvslggateasiwsi
WP_077855152.1   nklsssi---kid-------------------------------------------snesiliqnvnkensnnkfdslrlimlsgdyislnlpgkiksifqnaetislggateaaiwsi
WP_090670142.1   rpeyaag------------et-----------------------------------vretaaavtaasgnrhkerlslrlvllsgdwiplrlpqnikhhfagaevislggateasiwsi
WP_084669449.1   sneflnv------------ql------------------------------------neseigikraereqkhvnsslrvvmlsgdwiplslpniiknrfehanvyslggateaaiwsi
WP_083803565.1   apemieti--tvnrlesgpae----------------qtve-------------qk-----teetpsfragqwdaaslrlvllsgdwiplhlagqiktacpnaqiislggateasiwsi
WP_076252863.1   smeewssrny-irqnps-vsvsygenpmaadptllsersvavlgaqtaplanlniqssdie-r--avnitnegnhqsslrlillsgdwiplslpgkvrnlcpdasivslggateasiwsi
WP_105407286.1   pkedwirhesdirrnfsasetrygqstea-vqsertertiavlgeqsfsseqvngkgidva-rarvdntialsntktslrlvllsgdwipltlpdkirsiypdtsivslggateasiwsi
WP_013918046.1   gekr---------gerv-----------------------------------------------lqveeggalpslrlvllsgdwisvglpgrisrkfpdaaavslggateasiwsi
WP_088830566.1   aisl---------qesgitii---------s-----n-----e------------esgvstrg---lie-letkktslravllsgdyipvhlpakikssfngtkvislggateasiwsv
WP_081621455.1   esye---------eynqisete--------------n---------------------------nlke-frndfnnlrlvllsgdwiplklpekikkkfpssqvislggateaaiwsi
WP_152754018.1   diwn---------nfeeynskc-----alve---atvsidtld--------knymnenvtqqkvidikklsnkcaeslrlillsgdwipsnlpektrnhypnseiislggategsiwsi
WP_139487910.1   atkq---------aveqpsqa---------------------------------------tapspvpaseaqpvgtstlrlillsgdwipltlpqaakkqftkaqvislggateasiwsi
BmdB_A2          -------------------------------------------------------------------------HFENISLRLVLLSGDWIPLPLPAKINRHFPVADVISLGGATEASIWSI
BmdB_A2_BmdC     -------------------------------------------------------------------------HFENISLRLVLLSGDWIPLPLPAKINRHFPVADVISLGGATEASIWSI
                                                      Linker                                                                      A5

WP_118044189.1   yypigevdkkwksipygyplgnqkiyimntqghvlpvgvkgeiciagagvaegycndkdktekafvtypqygrvyrtgdfgilhkegyieflgrmdyqvklngyrvelqeienailqnpe
WP_077830295.1   yykinevkeewssipygvplsnqkiyilnedkmvlpidtigeiyiggvgvsdgyenniektkdafidhdslgriyktgdygimrkdgyieflgrkdnqvkingfrveleeienrlkkhqd
WP_077855152.1   yykincderydesipygyplqnqklyilnkdrklcppqvigeiaisgtgiadgyvndeektqksfifdeqlgrmyltgdygvltkkgyikflgrrdnqvkvngyrvelneiesKllqsqy
WP_090670142.1   yypiayedpkwksipygkplanqkfyvldqkgnpchlgvtgelyiggvglakgyrndpektgkafithpvlgslyrtgdygrmkedglieflgrmdhqvkirghrielgeiehgllkhpv
WP_084669449.1   yypviqeendwvsipygtplanqkfyvldykgdfchigvpgelyiggsglakgymndpektknafikhptlgmlyktgdygrlletgyieflgrkdnqvkirgyrielgeiencl ikqad
WP_083803565.1   yypiravktewssipygmplanqtiyimgldeelcpigtpgeimiggagvaeayindeqktqaafvvhpklgrlyktgdygifrpegyveflgrkdsqieigghrielgeitanleqnpn
WP_076252863.1   yypinsisdawnsipygyplanqtfhvldyklrpcpvdvpgemfiggvglanqyvndldktekafiqhpllgrlyrtgdhgvmrkegyieflgrkdnqikirghrvelgeieaifiqhha
WP_105407286.1   yysideissgwssipygyplanqmfyvldyklrscpmdvpgelyiggvgladhyvsdpektelafiqhpilgrlyrtgdhgvlrkegyieflgrkdnqikirghrvelgeieailnqhha
WP_013918046.1   gypiespadgwrsipygrplanqtihvlnyrmkpcpvdvpgelyiggmglaqeywqdedktnrafirhpelgrlyrtgdygvmrrggyvefigrrdqqvkirgyrvepgeieacllrhaa
WP_088830566.1   yypiektekgwtkipygmplgnqtihvlnysqqpcpvgvmgelyiggigvakeylkdeektkhafiqhkefgrlyrtgdygilhkdgtvefmgrkdhqlkiggyrvelgeiegrlleipe
WP_081621455.1   yypikeinnewksipygiplanhqfyvmnyerelcpvgikgdlyiggiglaegyyndeektqnafinhpefgkvyrtgdqgilnkngyieflgrndyqvkirghrielgeiehnilefgn
WP_152754018.1   fypikevksewnsipygyplqnqrfyvlnyenklcpigvkgelciagtglakkylndkektkkafidhnklgrlyktgdygimhkegyiefigrkdyqvkirgyrvemgeiehllmkhke
WP_139487910.1   yypieevkeewtsipygmplanqtfyvlnyeldlcpvgvrgelyiggvglaqqyfkdeektnhafithpkygrlyrtgdhgimrkegyiefmgrvdhqvkirgyrielgeiehnlldhea
BmdB_A2          YWPIEQVEANWKSIPYGKPLANQTYYVLNYDQKMCPVGVIGDLYIGGAGLAQGYLNDDQKTKDAFIMHPEFGPIYKTGDCGRMRPEGYIEFLGRQDYQVKIQGYRVELEEISHCLLTYPD
BmdB_A2_BmdC     YWPIEQVEANWKSIPYGKPLANQTYYVLNYDQKMCPVGVIGDLYIGGAGLAQGYLNDDQKTKDAFIMHPEFGPIYKTGDCGRMRPEGYIEFLGRQDYQVKIQGYRVELEEISHCLLTYPD
                                                                A6                         A7                 A8

WP_118044189.1   iencvvkmqkn-lkdnfliayytakhn-----vnetilktslrnllpsymipkyfmridkfylnsngkier
WP_077830295.1   ikecavvvq--geaktyicayyvsdkl-----vskeelekylrnsltdymipkyfvrlnsmpltfngkink
WP_077855152.1   ikdaavididddrkrkylvafvvsdse-----insdnirdslmeslpfymiprryfikvesipltpngkidk
WP_090670142.1   vkgaavvdvtetdgktylcayivsdqq-----miagelkehilkelpdymlpayfiqvsaipltsngkvdk
WP_084669449.1   iknvavidqankngkkylsafivsnsk-----inikelkekllkelpdymvpayfnqvneipltangkvdk
WP_083803565.1   takcvsvpytdtngieqicsyvvlkna-----vliselraylkerlpdymvparilevneipltdngklnt
WP_076252863.1   lkrvyvmdrqneqkkkylcayyvsdqe-----vsiaelreflisrlpeymvpsffirleaipltangkinr
WP_105407286.1   lkrvyvtdrlneqkkkylcayyvsdte-----vsitelraflisrlpeymvpsffirmeaipltangklnr
WP_013918046.1   irqalvvdridahsrkslcaylvasspasdpsleaagirhflqehlpeymvpehlvflnaipltpngkidr
WP_088830566.1   inhavvadftdgtnrkqlaayvvfhkr-----igfhvlreklmsslpyymipkyflevsnipvnyngkinr
WP_081621455.1   inraividskekegrkhlnayyssnld-----ispielrkyleqkipsymipsffvpikdipltpngkvdy
WP_152754018.1   innailidctdpqgkkslcayivsnkd-----ipvsvlreylsndlpvymipahfvylntipltpngkvdr
WP_139487910.1   vktavildktdangkkyicayyiphqe-----vstnelrehlqkripmymipsyfvpidhipltpngkvdr
BmdB_A2          VDQAVVIDQTDERGMKFLVGYVVAQQE-----IDEKALRKHLMEHLPEYMIPAHLVHLEQLPLTPNGKLDR
BmdB_A2_BmdC     VDQAVVIDQTDERGMKFLVGYVVAQQE-----IDEKALRKHLMEHLPEYMIPAHLVHLEQLPLTPNGKLDR
                                                                A9                A10
```

b

```
IndC_a8-ox-a9_a10    GRADNQVKLRGYRVELDEIRQTVETHDWVRAAAVLLRDDATTGFQNLVAFVELNPKEA-------ALMDQGNHGSHHQSKAS----RLQVRAQLAHPGCRDDADLAGRAAIDLPGAEATP   109
EpoB_a8-ox-a9_a10    GREDNQIKLRGYRVELGEIEETLKSHPNVRDAVIVPVGNDAAN-KLLLAYVVPEGTRRRAAEQDASLKTERVDARAHAAKADGLSDGERVQFKLARHGLR--RDLDGKPVVDLTGLVPRE   117
                     ** ***:*********.**.:*:::*  ** *.::  .: ::. : *:*:*  : ..        :*  :  ..  * :**.      :*: :**: * *   ** *: .:** *
                          A8 motif                        Insertion point|

IndC_a8-ox-a9_a10    GQRALAFSRKTYRFYEGSPVTRDDILHLLGPRPRPRPSARTSDIVGRDELGTILRNFGRHLSDQRLLPKYAYASPGSLYATQLYVE--IGGGHDVPAGLYYYHPLHHRLVLVGPASETE-   226
EpoB_a8-ox-a9_a10    AGLDVYARRRSVRTFLEAPIP-------------------------FVEFGRFLSCLSSVEPDGAALPKFRYPSAGSTYPVQTYAYAKSGRIEGVDEGFYYYHPFEHRLLKVSDHG-IER   211
BmdC_Ox                   RRSCRRFDMKTPVS-------------------------FATFSNLLSSLK---QRKEDKILYNYASAGGLYPIDVFVYVKPRRVEGVKAGFYYFNPADHSLVLVNNIDQVIK   85
                     .    :    *    *    :*:                              :. :*   :              : * * *. *   : :.       ..*  *:**::* .* *: *.   .

IndC_a8-ox-a9_a10    ------------TSPVRIHFLGKHGAIEPVYRNNVREVLEIEAGHMVGLFEEVLPAHGLRIAAAAYQPAVRHRLDCAPEDHYLGSFDLLPQARGASEDTDTLDIYVQAHSTRIEGLPPGQ   334
EpoB_a8-ox-a9_a10    GAHVPQNFDVFDEAAFGLLFVGRIDAIESLYGSLSREFCLLEAGYMAQLLMEQAPSCNIGVCP-------------------VGQFDFEQVRP--VLDLRHSDVYV-------HGMLG--   301
BmdC_Ox              DDHELINQDIFAQSAFSVYLVYNARASMPKYGAAGYFYACIEAGIITATLNMVAEDLNVGLCS-------------------IGHMNFEEIQT--FLKLEDHQVIL-------HAI-E--   174
                                  : . : :: .   *     *           :*** :.  :         .: :.                   :* :::          .     :: :       ..:

IndC_a8-ox-a9_a10    YRYTGAGLVRIGDDVILKKHVIAINQRVYERSDFGISLVATGSASWRRYLDLGRGLQRLQMNDLHLGFMSSGYSSKSGNDLPSAKRLGRILADGGLPAGPSYFCVGGRVSDA---QWRGE   451
EpoB_a8-ox-a9_a10    ----------------------------------------------------------------------------------------------GRVDPRQFQVCTLGQDSSPRRATTRGA   328
BmdC_Ox              ----------------------------------------------------------------------------------------------GGLKIDG--------------------   181
                                                                                                                   * :

IndC_a8-ox-a9_a10    DMKEDVVHMQGPAELIKEDLAALLPRYMLPNRIVVLDRLPQTANGKID      499
EpoB_a8-ox-a9_a10    P----PGRDQHFADILRDFLRTKLPEYMVPTVFVELDALPLTSNGKVD      372
                             : *   *::::: * :  **.**:*. :* ** ** *:***:*
                  Insertion point|         A9 motif          A10 motif
```

c

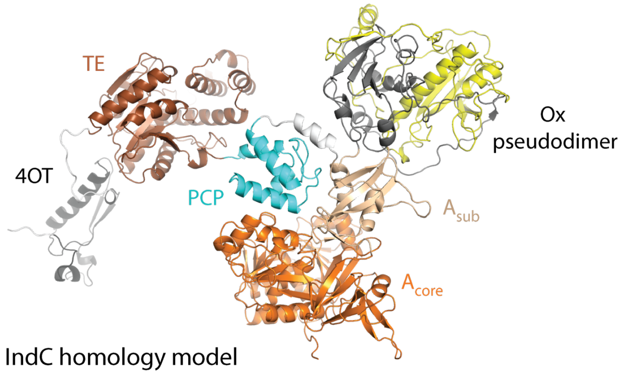

Supplementary Figure 7: **Embedded oxidase domains in NRPSs**

a. Bioinformatic analysis and alignment of BmdB_A$_2$ with the thirteen NRPS/PKS systems including oxidase insertions within their A domains. The insertion points of these oxidases are within the same loop of the A domains and correspond to the residues Ser1534 and His1535 in BmdB. The conserved motifs in adenylation domains are highlighted in yellow. **b**. Sequence alignment of the embedded oxidases in IndC and EpoB with BmdC reveal that the oxidase inserted in IndC is much larger than that of EpoB and BmdC. These insertion points are highlighted in red **c**. A homology model of indigoidine synthetase shows that it contains a pseudo-dimeric Ox domain (one half of the pseudo-dimer colored in dark gray and the other half of the pseudo-dimer colored in yellow) inserted into the Asub domain (light orange), between motifs A8 and A9. This position of Ox would not allow the interactions analogous those seen in BmdC Ox: BmdB_A$_2$. The homology model was constructed using Robetta[10], SWISS-MODEL[11] and manual modelling. 4OT: terminal tautomerization domain (gray). The TE domain of the homology model is depicted in brown.

Supplementary References

1 Bloudoff, K., Fage, C. D., Marahiel, M. A. & Schmeing, T. M. Structural and mutational analysis of the nonribosomal peptide synthetase heterocyclization domain provides insight into catalysis. *Proc Natl Acad Sci U S A* **114**, 95-100 (2017).

2 Contestabile, R. *et al.* l-Threonine aldolase, serine hydroxymethyltransferase and fungal alanine racemase. A subgroup of strictly related enzymes specialized for different functions. *Eur J Biochem* **268**, 6508-6525 (2001).

3 Maier, T. H. Semisynthetic production of unnatural L-alpha-amino acids by metabolic engineering of the cysteine-biosynthetic pathway. *Nat Biotechnol* **21**, 422-427 (2003).

4 Phillips, R. S. Synthetic applications of tryptophan synthase. *Tetrahedron-Asymmetr* **15**, 2787-2792 (2004).

5 di Salvo, M. L. *et al.* Alanine racemase from *Tolypocladium inflatum*: a key PLP-dependent enzyme in cyclosporin biosynthesis and a model of catalytic promiscuity. *Arch Biochem Biophys* **529**, 55-65 (2013).

6 Soo, V. W., Yosaatmadja, Y., Squire, C. J. & Patrick, W. M. Mechanistic and evolutionary insights from the reciprocal promiscuity of two pyridoxal phosphate-dependent enzymes. *J Biol Chem* **291**, 19873-19887 (2016).

7 Regni, C. A. *et al.* How the MccB bacterial ancestor of ubiquitin E1 initiates biosynthesis of the microcin C7 antibiotic. *EMBO J* **28**, 1953-1964 (2009).

8 Tanner, J. J., Tu, S. C., Barbour, L. J., Barnes, C. L. & Krause, K. L. Unusual folded conformation of nicotinamide adenine dinucleotide bound to flavin reductase P. *Protein Sci* **8**, 1725-1732 (1999).

9 Ghilarov, D. *et al.* Architecture of microcin B17 synthetase: An octameric protein complex converting a ribosomally synthesized peptide into a DNA gyrase poison. *Mol Cell* **73**, 749-762 (2019).

10 Yang, J. Y. *et al.* Improved protein structure prediction using predicted interresidue orientations. *Proc Natl Acad Sci U S A* **117**, 1496-1503 (2020).

11 Waterhouse, A. *et al.* SWISS-MODEL: Homology modelling of protein structures and complexes. *Nucleic Acids Res* **46**, W296-W303 (2018).
