# Supplementary material for: Structures and function of a tailoring oxidase in complex with a nonribosomal peptide synthetase module
Source: Nat Commun. 2022 Jan 27;13:548. doi: 10.1038/s41467-022-28221-y (PMC8795117; doi:10.1038/s41467-022-28221-y)
Supplement: Supplementary file 3 — Reporting Summary [file 41467_2022_28221_MOESM3_ESM.pdf]

nature research

Corresponding author(s): Martin Schmeing

Last updated by author(s): Nov 23, 2021

Reporting Summary

Nature Research wishes to improve the reproducibility of the work that we publish. This form provides structure for consistency and transparency in reporting. For further information on Nature Research policies, see our Editorial Policies and the Editorial Policy Checklist.

Please do not complete any field with "not applicable" or n/a. Refer to the help text for what text to use if an item is not relevant to your study.
For final submission: please carefully check your responses for accuracy; you will not be able to make changes later.

Statistics

For all statistical analyses, confirm that the following items are present in the figure legend, table legend, main text, or Methods section.

n/a	Confirmed	
☐	☒	The exact sample size (*n*) for each experimental group/condition, given as a discrete number and unit of measurement
☐	☒	A statement on whether measurements were taken from distinct samples or whether the same sample was measured repeatedly
☒	☐	The statistical test(s) used AND whether they are one- or two-sided<br>*Only common tests should be described solely by name; describe more complex techniques in the Methods section.*
☒	☐	A description of all covariates tested
☐	☒	A description of any assumptions or corrections, such as tests of normality and adjustment for multiple comparisons
☐	☒	A full description of the statistical parameters including central tendency (e.g. means) or other basic estimates (e.g. regression coefficient) AND variation (e.g. standard deviation) or associated estimates of uncertainty (e.g. confidence intervals)
☒	☐	For null hypothesis testing, the test statistic (e.g. *F*, *t*, *r*) with confidence intervals, effect sizes, degrees of freedom and *P* value noted<br>*Give P values as exact values whenever suitable.*
☒	☐	For Bayesian analysis, information on the choice of priors and Markov chain Monte Carlo settings
☒	☐	For hierarchical and complex designs, identification of the appropriate level for tests and full reporting of outcomes
☒	☐	Estimates of effect sizes (e.g. Cohen's *d*, Pearson's *r*), indicating how they were calculated

Our web collection on statistics for biologists contains articles on many of the points above.

Software and code

Policy information about availability of computer code

Data collection	Cryo-EM data: SerialEM v3.8 for automated data collection. X-ray data: Synchrotrons - Advanced Photon Source (APS) and Canadian Light Source (CLS). Mass Spectrometry data: . ESI-MS was performed with an in-line Bruker amaZon speed ETD ion trap mass spectrometer. Isothermal calorimetry (ITC) data was collected with MicroCal iTC 200 (GE Healthcare).
Data analysis	Crystallography data were processed and analyzed using iMosflm, HKL2000, CCP4 Suite (including AIMLESS, Coot, MORDA, Sketcher), Phenix v1.15.3 (Including Phaser, Autobuild, REEL), PyMOL v2.4.1. EM data were processed using CryoSPARC2. Bioinformatics: CLUSTAL OMEGA, BLASTP, Robetta. Isothermal calorimetry (ITC) data was analyzed with Microcal Origin 7.0 software package. Additional linear regression analysis was performed with GraphPad Prism v6.0.0.

For manuscripts utilizing custom algorithms or software that are central to the research but not yet described in published literature, software must be made available to editors and reviewers. We strongly encourage code deposition in a community repository (e.g. GitHub). See the Nature Research guidelines for submitting code & software for further information.

Data

Policy information about availability of data

All manuscripts must include a data availability statement. This statement should provide the following information, where applicable:

- Accession codes, unique identifiers, or web links for publicly available datasets
- A list of figures that have associated raw data
- A description of any restrictions on data availability

Structure coordinates for this study have been deposited in the PDB under the accession codes: 7ly4, 7ly5, 7ly6 and 7ly7. The cryo-EM maps can be retrieved from the EMDB under the accession codes: EMD-23587 and EMD-23588. The source data for all figures with associated raw data have been provided.

Field-specific reporting

Please select the one below that is the best fit for your research. If you are not sure, read the appropriate sections before making your selection.

☒ Life sciences ☐ Behavioural & social sciences ☐ Ecological, evolutionary & environmental sciences

Life sciences study design

All studies must disclose on these points even when the disclosure is negative.

Sample size	No sample size calculations were performed. In general, experiments in the study were done in triplicate as a minimal number to account for the experimental error.
Data exclusions	There is no data excluded from the analysis, except for one ITC run due to hardware failure.
Replication	Quantitative data was performed minimally in triplicate. All replicates were successful, except for one ITC run due to hardware failure.
Randomization	N/A - all the samples in their respective experiments were subjected to the same in vitro conditions. There were no animal or human subjects/groups in this study for randomization.
Blinding	N/A - There were no animal or human subjects involved in the experiments presented in this manuscript to need a blinding experiment.

Reporting for specific materials, systems and methods

We require information from authors about some types of materials, experimental systems and methods used in many studies. Here, indicate whether each material, system or method listed is relevant to your study. If you are not sure if a list item applies to your research, read the appropriate section before selecting a response.

Materials & experimental systems

n/a	Involved in the study	
☒	☐	Antibodies
☒	☐	Eukaryotic cell lines
☒	☐	Palaeontology and archaeology
☒	☐	Animals and other organisms
☒	☐	Human research participants
☒	☐	Clinical data
☒	☐	Dual use research of concern

Methods

n/a	Involved in the study	
☒	☐	ChIP-seq
☒	☐	Flow cytometry
☒	☐	MRI-based neuroimaging

This checklist template is licensed under a Creative Commons Attribution 4.0 International License, which permits use, sharing, adaptation, distribution and reproduction in any medium or format, as long as you give appropriate credit to the original author(s) and the source, provide a link to the Creative Commons license, and indicate if changes were made. The images or other third party material in this article are included in the article's Creative Commons license, unless indicated otherwise in a credit line to the material. If material is not included in the article's Creative Commons license and your intended use is not permitted by statutory regulation or exceeds the permitted use, you will need to obtain permission directly from the copyright holder. To view a copy of this license, visit http://creativecommons.org/licenses/by/4.0/
