# Supplementary material for: Structures and function of a tailoring oxidase in complex with a nonribosomal peptide synthetase module
Source: Nat Commun. 2022 Jan 27;13:548. doi: 10.1038/s41467-022-28221-y (PMC8795117; doi:10.1038/s41467-022-28221-y)

This word file contains all the uncropped images of the gels (initial photo taken) presented in the manuscript, separated by figure number.

**Supplementary Figure 1a**


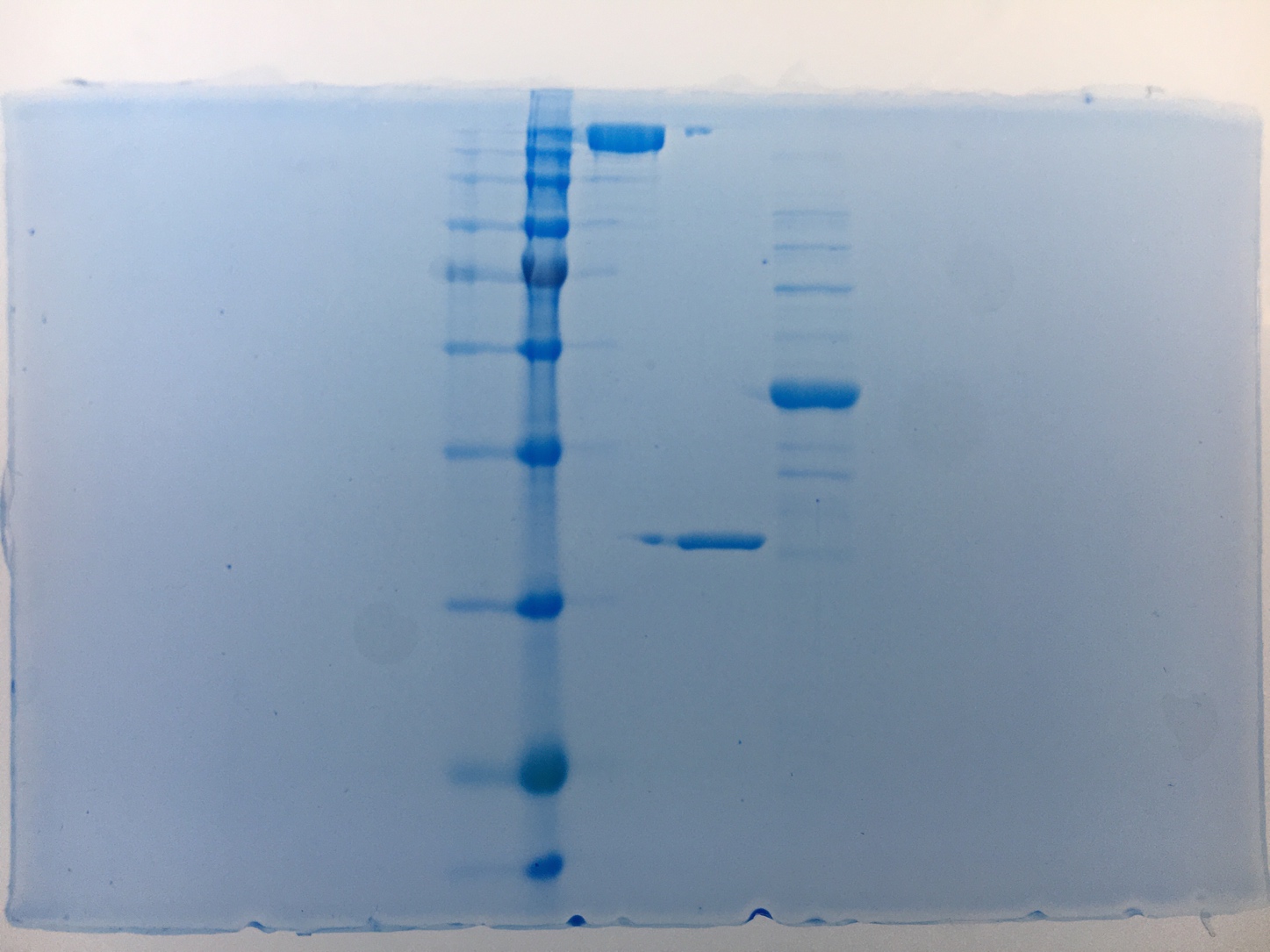


**Supplementary Figure 4b**

**This is the 12% gel**


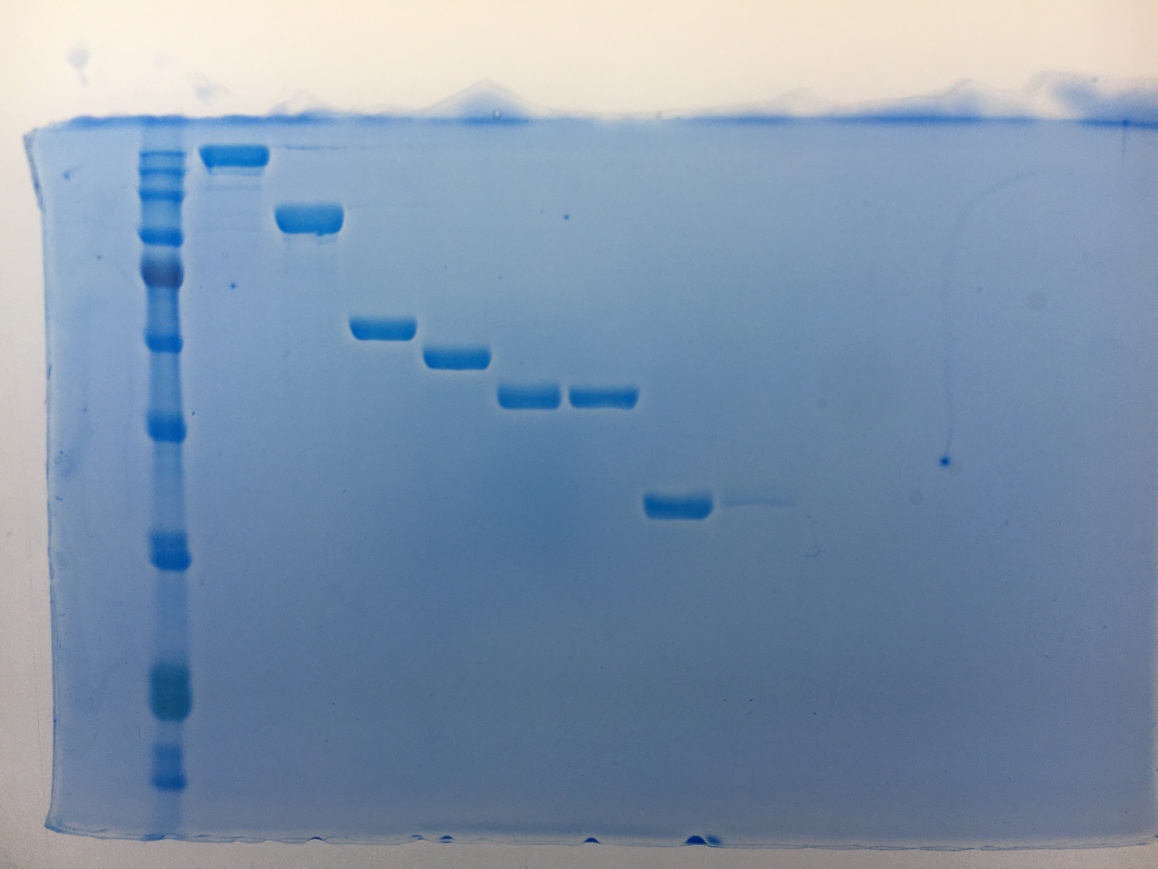


**This is the 15% gel**


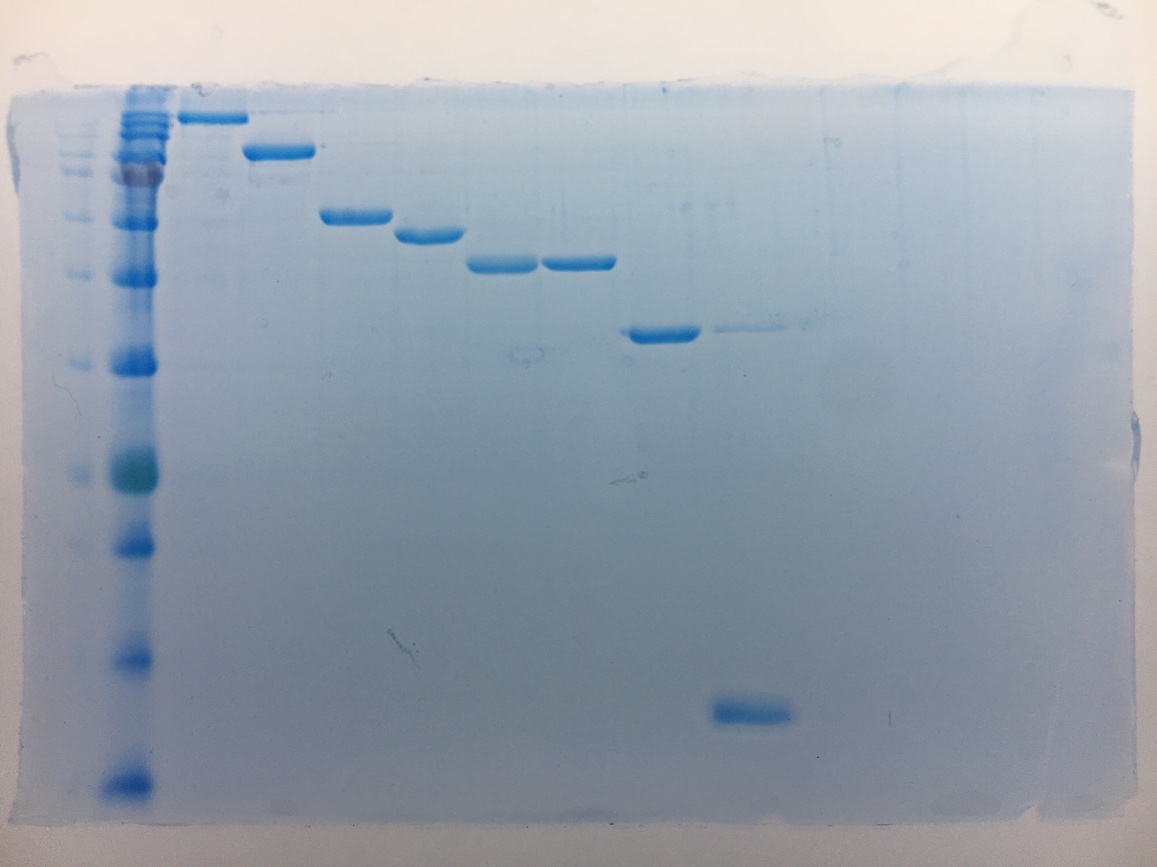


**Supplementary Figure 5c**


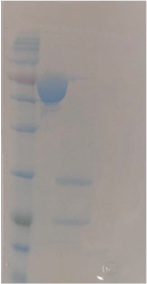

Supplement: Supplementary file 4 — Source Data [file 41467_2022_28221_MOESM4_ESM.zip › Source_data/Consolidated_uncropped_gels.docx]
