# Supplementary material for: Structures and function of a tailoring oxidase in complex with a nonribosomal peptide synthetase module
Source: Nat Commun. 2022 Jan 27;13:548. doi: 10.1038/s41467-022-28221-y (PMC8795117; doi:10.1038/s41467-022-28221-y)
Supplement: Supplementary file 4 — Source Data [file 41467_2022_28221_MOESM4_ESM.zip › Source_data/Main_Figure_3f/kD_VALUES.docx]

this is for the first one (replicate)

this is for the SECOND one (replicate)

For the “NEW” file

this is for the third replicate (labelled AS "4")
